# Intergenerational Mentoring on Health Promotion to Improve Healthy Lifestyles Among Latino Families

**DOI:** 10.1093/geroni/igaa057.1077

**Published:** 2020-12-16

**Authors:** Karen Kopera-Frye, Ashley Graboski-Bauer, Ana Luisa Gonzalez-Marquez

**Affiliations:** New Mexico State University, Las Cruces, New Mexico, United States

## Abstract

New Mexico now has the 32nd highest adult obesity rate; approximately one-third are Latinos. Obesity rates in NM for children aged 2-11 years range from 11 – 14%. Mentoring as a health promotion or intervention strategy has become widespread. However, few programs have focused on several generations reciprocally influencing each other in healthy behaviors. Project I’M HIP (Intergenerational Mentoring Health Information Pathways)’s goals included: Providing an innovative, multigenerational educational program to promote greater maternal, child, and grandparent well-being, healthier lifestyle behaviors, and support continued healthy home environments by empowering the families with knowledge. Three cohorts of 30 families (1 parent, 1 child, 1 grandparent/other relative) were recruited for Program I’M HIP. This Program utilized culturally sensitive Evidence-Based Programs (EBPs). Monthly educational sessions focused on physical activity and adapting meals to be healthy. Project outcomes included exercise frequency, Body Mass Index (BMI), and a knowledge quiz assessing healthy meal facts, exercise knowledge via a 10-item quiz; all assessments pre- and post-program. Program outcomes included: 100% of the parents shared at least 1 fact on nutrition or exercise with other relatives, thus affecting another household; paired t-test analyses revealed significant changes in knowledge quiz total scores (t 70 = 5.03, p < .0001), increased exercise frequency (t 72 = 2.106, p < .05); no significant change in BMI from pre- to post-assessments; and children corrected their parents on proper diet; all demonstrating the reciprocal mentoring effects of parent, child, and other relative on health behaviors.

